# Nucleobase and Nucleoside Analogues: Resistance and Re-Sensitisation at the Level of Pharmacokinetics, Pharmacodynamics and Metabolism

**DOI:** 10.3390/cancers10070240

**Published:** 2018-07-23

**Authors:** Nikolaos Tsesmetzis, Cynthia B. J. Paulin, Sean G. Rudd, Nikolas Herold

**Affiliations:** 1 Childhood Cancer Research Unit, Department of Women’s and Children’s Health, Karolinska Institutet, 171 77 Stockholm, Sweden; nikolaos.tsesmetzis@ki.se; 2 Science for Life Laboratory, Department of Oncology-Pathology, Karolinska Institutet, 171 65 Stockholm, Sweden; cynthia.paulin@scilifelab.se; 3 Paediatric Oncology, Theme of Children’s and Women’s Health, Karolinska University Hospital Solna, 171 76 Stockholm, Sweden

**Keywords:** chemoresistance, sensitisation, novel therapy, combination therapy, antimetabolites, nucleoside analogues, nucleobase analogues, cytarabine, gemcitabine, clofarabine, fludarabine, nelarabine, cladribine, 5-fluorouracil, capecitabine, SAMHD1, ribonucleotide reductase, precision medicine

## Abstract

Antimetabolites, in particular nucleobase and nucleoside analogues, are cytotoxic drugs that, starting from the small field of paediatric oncology, in combination with other chemotherapeutics, have revolutionised clinical oncology and transformed cancer into a curable disease. However, even though combination chemotherapy, together with radiation, surgery and immunotherapy, can nowadays cure almost all types of cancer, we still fail to achieve this for a substantial proportion of patients. The understanding of differences in metabolism, pharmacokinetics, pharmacodynamics, and tumour biology between patients that can be cured and patients that cannot, builds the scientific basis for rational therapy improvements. Here, we summarise current knowledge of how tumour-specific and patient-specific factors can dictate resistance to nucleobase/nucleoside analogues, and which strategies of re-sensitisation exist. We revisit well-established hurdles to treatment efficacy, like the blood-brain barrier and reduced deoxycytidine kinase activity, but will also discuss the role of novel resistance factors, such as SAMHD1. A comprehensive appreciation of the complex mechanisms that underpin the failure of chemotherapy will hopefully inform future strategies of personalised medicine.

## 1. Introduction

**Figure 1:**
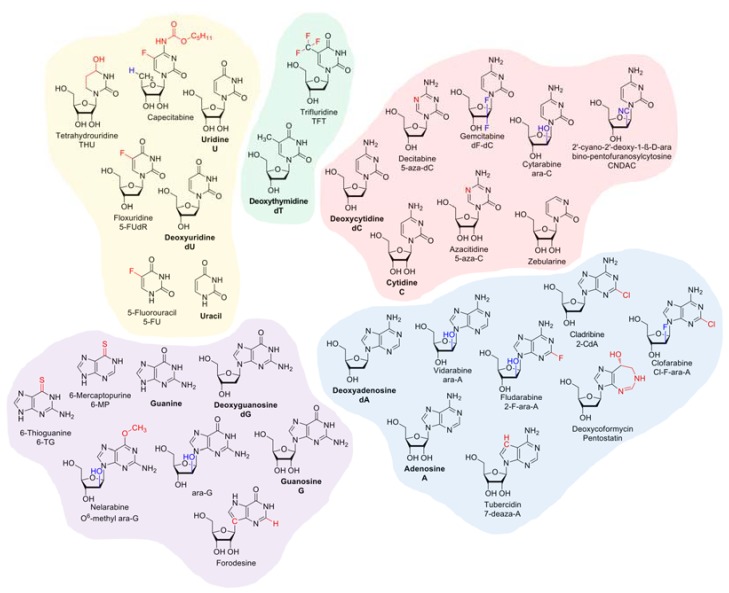
Structures of nucleobase and nucleoside analogues discussed in this review. Endogenous nucleobase/nucleosides are labelled in bold and synthetic analogues with sugar-modifications (indicated in blue) or base-modifications (indicated in red) are shown.

Since metastatic potential is a hallmark of cancer [1], management of malignant disease usually requires systemic treatment in order to prevent and treat tumour spread. Combination chemotherapy still constitutes the current paradigm to achieve systemic disease control in clinical oncology, even though immunotherapeutic approaches are becoming a viable complement at least for a subset of patients [2,3]. Antimetabolites were the first class of cytotoxic drugs systematically tested in clinical trials that elicited complete clinical responses as monotherapies, albeit with inevitable relapse [4]. Even though this review will–with few exceptions–mainly focus on monotherapy, it is important to keep in mind that, empirically, combination of chemotherapeutic agents is a *sine qua non* for the cure of the vast majority of cancers. On the other hand, a reductionist understanding of the mechanisms underlying the insufficiency of monotherapies is a prerequisite to rationally improve existing therapy modalities. This review aims to give an overview of the current understanding of chemoresistance, but will exclusively focus on nucleoside and nucleobase analogues (Figure 1), the major subgroup of antimetabolites.

Antimetabolites can be grouped into folate antagonists and nucleobase/nucleoside analogues. Due to their structural similarity, folate antagonists, or antifolates, either inhibit conversion of dihydrofolate to tetrahydrofolate by targeting dihydrofolate reductase (DHFR) or directly inhibit one or more of the enzymes that require tetrahydrofolate as a co-factor, e.g., phosphoribosylglycinamide formyltransferase (GARFT) and thymidylate synthase (TS); key enzymes in de novo synthesis of nucleic acid precursors (discussed in more detail below) [5,6]. Antifolates are, however, not within the scope of the current review article; comprehensive reviews are to be found elsewhere [7,8].

**Figure 2:**
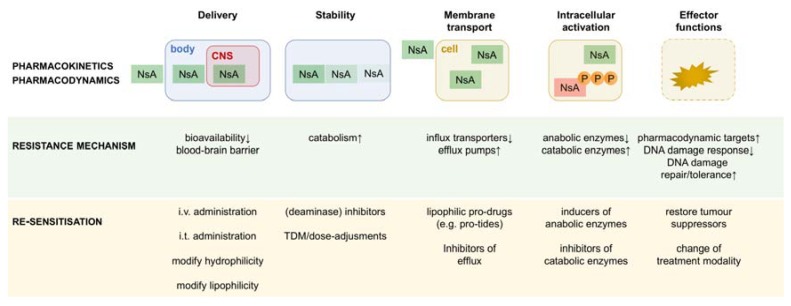
Schematic representation of the levels of resistance to nucleobase/nucleoside analogues. Resistance to nucleobase/nucleoside analogues can occur at the pharmacokinetic levels of delivery (e.g., due to the blood-brain barrier), stability (e.g., due to plasmatic catabolic activity), membrane transport (e.g., due to down-regulation of influx transporters), and intracellular activation (due to an imbalance in anabolic and catabolic enzymes). Further downstream, pharmacodynamic resistance can occur (e.g., due to overexpression of drug targets). Drug efficacy critically depends on the underlying tumour biology that determines the general susceptibility to cytotoxicity (for details, see text). Examples for re-sensitisation strategies are given. NsA, nucleoside analogue; TDM, therapeutic drug monitoring; CNS, central nervous system.

Nucleobase and nucleoside analogues exert their cytotoxic effects by mimicking endogenous nucleosides (and following phosphorylation, nucleotides). This can either be mediated by enzyme inhibition or by substituting endogenous nucleoside species as substrates, leading to DNA and RNA damage and interference with DNA methylation. Nucleoside analogues have to reach tumour sites, niches and sanctuaries at sufficient concentration (delivery) and in a non-degraded form (stability), be taken up into the cancer cell (usually by nucleoside transport proteins), and be converted into their active metabolites (activation) before they can hit their molecular target (pharmacodynamic activity) (Figure 2 and Table 1). Both disease- and patient-specific treatment failure to one or several nucleobase or nucleoside analogues can be caused at one or more of these steps. Strategies to rationally improve antimetabolic treatments have thus to take into account all of these mechanisms and assess their relative contribution.

## 2. Overview on the Pharmacodynamics of Nucleobase and Nucleoside Analogues

**Figure 3:**
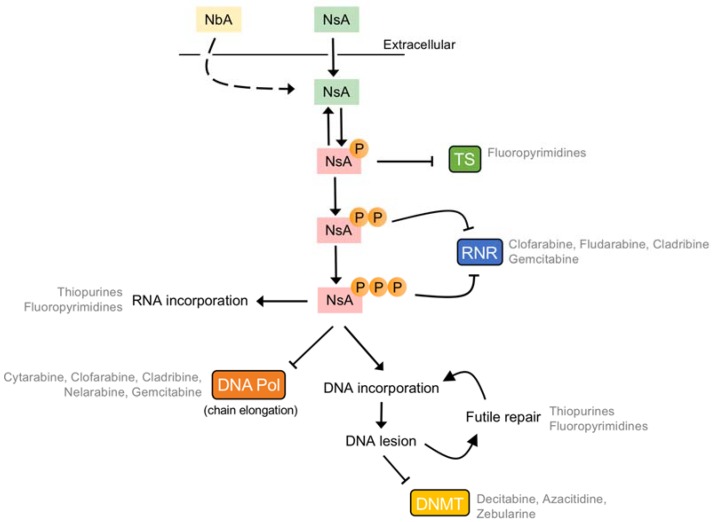
Overview on the pharmacodynamics of nucleobase/nucleoside analogues. Nucleobase (NbA, light yellow) and nucleoside analogues (NsA, light green) are metabolised intracellularly to produce their active metabolites (NsA, light red), be it the mono- (P), di- (PP) and triphosphate (PPP) species. These can inhibit key enzymes in DNA precursor metabolism, such as thymidylate synthase (TS) or ribonucleotide reductase (RNR), or be incorporated into nucleic acids. Here, these analogues can perturb DNA synthesis by DNA polymerases (DNA Pol) at the extension step, or the resultant genomic lesions can inhibit other enzymes, such as DNA methyltransferases (DNMTs), or lead to futile DNA repair cycles.

All nucleobase and nucleoside chemotherapeutics are pro-drugs, requiring chemical modification, typically multiple, such as sequential phosphorylation, to generate their active metabolites, even though nucleoside analogues themselves (i.e., no phosphates) can have inhibitory activities towards enzymes in vitro [138]. Because of this, these compounds interact with many cellular targets and perturb as many cellular processes, with an obvious focus upon nucleotide and nucleic acid metabolism. Thus, the mode of action of these compounds is multi-faceted. However, following decades of research, principal mechanisms of action have come into focus for the majority of these compounds (Figure 3), which are discussed below.

One common target of this family of compounds is DNA synthesis, as the triphosphate forms can compete with their endogenous dNTP counterparts for utilisation by DNA polymerases, thus being incorporated into the DNA molecule. For some of these analogues, owing to the replacement of the deoxyribose sugar with arabinose or another modified sugar moiety, these compounds perturb the DNA synthetic reaction, typically the extension step from the mis-incorporated analogue. Although sometimes referred to as chain terminators, these sugar modified analogues should rather be considered as ‘relative’ chain terminators. Extension from these termini is still possible, and occurs in cells and in vitro, unlike antiviral dideoxynucleosides, which lack the 3′-OH required for DNA synthesis, and are thus *bona fide* chain terminators. For instance, the triphosphate metabolite of cytarabine (ara-C) is readily incorporated into DNA by cellular replicases in vitro, with an efficiency comparable with dCTP. However, the extension from the mis-incorporated ara-CMP terminus by these enzymes occurs with a significantly reduced efficiency [139,140,141,142]. In line with this, ara-C treated cells quickly accumulate genomic ara-CMP, and this coincides with decreased DNA synthesis and activation of the intra-S-phase checkpoint [117,143], indicating that it is likely this delayed extension, slowing nascent chain synthesis, and the resulting replication fork stalling, that contributes to cancer cell death. This model is supported by a recent study that also highlights the critical role of polymerase proofreading in this process [142]. Although the extension step is perturbed, it still occurs, as evidenced by cells treated with tritiated ara-C incorporating the labelled analogue into growing DNA fragments [144,145,146,147]. Other sugar-modified analogues, such as the triphosphate of fludarabine (2-F-ara-ATP), function as stronger chain terminators, evidenced by cells incubated with tritiated fludarabine accumulating this analogue at DNA termini [148]. Cellular effects upon DNA synthesis are likely relative to the abundance of triphosphates present. For instance, in cells incubated with low concentrations of tritiated clofarabine (Cl-F-ara-A), this analogue can be detected at inter-nucleotide linkages. This is consistent with extension from the mis-match and continued DNA synthesis occurring, whilst with high concentrations, this analogue is primarily detected at DNA termini [149], consistent with chain termination. In contrast to these drugs, gemcitabine (dF-dC) triphosphate (dF-dCTP) possesses a unique chain termination activity, in which primer elongation is inhibited following addition of one nucleotide after the mis-incorporated dF-dCMP [150]. This has been termed “masked chain termination” as it would make the mis-incorporated analogue refractory to nuclease-mediated excision [151]. In addition to these mechanisms, the DNA incorporation of the monophosphate of 2′-cyano-2′-deoxy-1-β-d-arabinopentofuranosylcytosine (CNDAC), CNDACMP, leads to chain termination due to a process called beta-elimination owing to the high electrophilicity of the cyano group at the 2′-carbon [152], resulting in a single-strand DNA break, possibly triggering cell-cycle arrest in the G2-phase [153].

Whilst the analogues discussed above have an immediate effect on replicating cells, owing to their modified sugar moieties perturbing DNA synthesis, others, such as decitabine and thiopurines, which lack sugar modification, have a comparatively delayed mechanism of action. The triphosphate metabolite of thiopurines, 6-thio-dGTP, is readily incorporated into the genome, reported to replace canonical guanine nucleotides by up to 0.1% [154,155,156,157], and this incorporation itself is neither toxic nor mutagenic [155,158,159]. However, toxicity is triggered when genomic 6-thio-dGMP is non-enzymatically methylated by S-adenosylmethionine (SAM), as following a round of DNA replication, this will result in a 6-MeThio-dG:dT mis-pair [160,161]. In this scenario, the mis-incorporated dT opposite the thioguanine lesion is now recognised as an error by mismatch repair (MMR), a DNA repair pathway that ensures faithful genome duplication, and is subsequently excised from the nascent DNA strand [161], but as the correct pairing cannot be made, this leads to futile repair cycles [162,163,164]. The resulting unrepaired gaps will then be converted into cytotoxic DNA breaks upon encountering a replication fork in the next S-phase [158,163,165]. It’s interesting to note, that, whilst thiopurine cytotoxicity is MMR-dependent, the proficiency of this repair pathway can vary widely amongst T-cell acute lymphoblastic leukaemia (T-ALL) patients [166,167], all of which receive thiopurines during maintenance therapy. Whilst other mechanisms of action exist, including incorporation into RNA of the thiopurine ribonucleotide triphosphate metabolite (6-thio-GTP), perturbing mRNA transcription [168], and inhibition of Rac1 GTPase [169], perhaps the clinical combination of thiopurines with nucleotide biosynthesis inhibitors (such as methotrexate), further promotes 6-thio-dGTP mis-incorporation, overcoming the necessity of MMR. Like thiopurine triphosphates, decitabine triphosphate (5-aza-dCTP) can also be readily incorporated into DNA [170], and it is the resultant genomic 5-aza-dCMP that is responsible for this analogues therapeutic effect. When incorporated prior to dG, resulting in a 5-aza-dC-p-dG dinucleotide, this can be recognised by DNA methyltransferases (DNMTs), which typically methylate the 5-position of cytosines present in CpG dinucleotides to silence gene expression. However, resulting from the chemistry of the methyltransferase reaction with 5-aza-dC, DNMT becomes covalently linked to DNA, generating a DNA-protein cross-link [171,172,173]. This can lead to two possible outcomes, which may be dependent upon the amount of genomic 5-aza-dCMP. Lower levels of genomic 5-aza-dCMP will lead to tolerable levels of DNMT-DNA crosslinks, which deplete soluble DNMT pools, resulting in global reduction of genomic methylation levels and augmentation of gene expression patterns [174]. However, high genomic 5-aza-dCMP will result in high amounts of DNMT-DNA crosslinks which present a physical block to the DNA synthesis machinery and will result in frequent replication-dependent cytotoxic DNA breaks [175,176], which likely affects cell fitness more immediately than alterations in gene expression patterns. Another DNMT inhibitor following DNA incorporation of its 2′-deoxy variant is the cytidine analogue and cytidine deaminase inhibitor zebularine [62].

In addition to mis-incorporation into DNA and perturbation of DNA metabolism, inhibition of nucleotide metabolism enzymes can also be a key factor in the cytotoxic mechanism of nucleobase and nucleoside analogues. A key enzyme in de novo dNTP biosynthesis is ribonucleotide reductase (RNR), which reduces nucleoside diphosphates (NDPs) to deoxyNDPs (dNDPs), and several of these analogues efficiently target this enzyme. The diphosphate form of gemcitabine (dF-dCDP) is a substrate analogue of RNR [177], demonstrated to be an irreversible suicide inhibitor, becoming covalently bound to the large subunit RRM1 [178]. In contrast, metabolites of purine nucleoside analogues clofarabine (Cl-F-ara-A), fludarabine (2-F-ara-A) and cladribine (2-CdA), all inhibit RNR, but in a reversible manner. In this case, both the di- and triphosphate forms are responsible for RNR inhibition, interacting with the catalytic and allosteric sites respectively, altering the quaternary structure and promoting formation of persistent RRM1 hexamers [179,180,181]. The dual activity of these purine analogues, and gemcitabine, as RNR and DNA synthesis inhibitors, leads to a mechanism of self-potentiation, in that these analogues inhibit de novo dNTP synthesis that in-turn reduces the level of competing endogenous dNTPs, thus promoting their mis-incorporation into DNA. The mechanism of action of thiopurines has also been linked to inhibition of nucleotide biosynthesis, in particular inhibition of the key purine biosynthesis enzyme phosphoribosyl pyrophosphate amidotransferase (PPAT) by the metabolite methylthioinosine monophosphate [182]. However, the contribution of this to cytotoxicity remains unclear [158], with long-existing evidence arguing the contrary [183].

Another important DNA metabolism target of nucleobase and nucleoside therapeutics is thymidylate synthase (TS), which is irreversibly inhibited by fluoropyrimidines (e.g., 5-FU, floxuridine, 5-FU pro-drug capecitabine) [184,185]. TS catalyses the reductive methylation of deoxyuridine monophosphate (dUMP) to deoxythymidine monophosphate (dTMP), using the folate 5,10-methylenetetrahydrofolate (CH_2_THF) as the methyl donor [186]. This provides the sole source of de novo dTMP ultimately required for DNA synthesis and is thus considered one of the few metabolic bottlenecks for the synthesis of DNA [185]. The monophosphate form of fluoropyrimidines, 5-F-dUMP, is a substrate analogue, competing with dUMP, and thus binds to the catalytic site of TS resulting in a stable ternary complex with the enzyme and CH_2_THF. This prevents normal substrate binding and thus inhibits the enzyme [187,188], which is the basis for enhanced 5-FU efficacy by addition of folinic acid [189]. In addition to 5-FU and derivatives, trifluridine (TFT), activated to TFT monophosphate by thymidine kinases (TKs), also inhibits TS [190]. TS inhibition depletes intracellular dTMP, which in turn leads to a shortage of dTTP (and due to various feedback mechanisms, also perturbs levels of other dNTPs), required for DNA synthesis [191,192,193]. In addition, dUMP pools become expanded, due to lack of TS activity turning over this metabolite, which can be phosphorylated to dUTP and mis-incorporated into DNA, along with the triphosphate form of fluoropyrimidines (5-F-dUTP and TFT triphosphate, TFT-TP) [194,195]. As 5-F-dUTP, TFT-TP and dUTP are readily incorporated into DNA, comparable to dTTP [195,196,197], a mechanism of self-potentiation is also evident here. Unlike the sugar modified analogues, and much like the thiopurines and decitabine, the use of fluoropyrimidines (or uracil) in DNA synthesis is not cytotoxic itself, however the resultant genomic (fluoro)uracils and trifluorothymines can promote mutagenesis and cytotoxic DNA lesions [195,198,199]. Accordingly, DNA repair pathways exist to remove these lesions from the genome, including MMR and base excision repair (BER) [200,201], the latter containing enzymes specifically evolved for the removal of genomic uracil and thymine mis-pairs, such as UNG, SMUG, TDG, and MDB4 [202,203]. However, the abasic site intermediate of BER can be more detrimental to the cell than the original DNA lesion, especially if they persist due to futile repair cycles as a result of expanded fluoro(uracil) pools, ultimately leading to DNA breaks [199]. In addition to inhibition of TS and incorporation into DNA, fluoropyrimidine ribonucleotide triphosphates (5-FUTP) can be incorporated into nascent RNA molecules, perturbing a number of processes, including ribosomal RNA maturation and mRNA splicing [204,205]. The importance of RNA incorporation in the mechanism of action of fluoropyrimidines is underscored by numerous examples of treatment with exogenous uridine protecting cells from 5-FU cytotoxicity [206,207]. In fact, uridine is an FDA-approved rescue-treatment for overdose or severe toxicity with 5-FU or capecitabine [208]. All of these mechanisms likely contribute to therapeutic efficacy of fluoropyrimidines, however, despite decades of research, the relative contribution is still a topic of much debate.

It should be noted that (deoxy)adenosine analogues have also been reported to bind to purinergic receptors and exert agonistic or antagonistic activity that can modulate down-stream signalling. This suggests that at least a portion of nucleoside analogue-mediated toxicity might be due to engagement of the extracellular portion of membrane proteins, even though the practical implications remain elusive [209,210,211,212]. It is tempting to speculate whether nucleoside analogue di- and triphosphates, released during cell death, could mimic the antiproliferative effects of ATP and UTP via P2Y receptor engagement in bystander tumour cells [213].

Other nucleoside analogues exist in the clinical or pre-clinical setting that cannot be discussed in detail here. Tubercidin, toyocamycin, sangivamycin and derivatives are bacteria-derived adenosine analogues with different effects on RNA and DNA, protein kinase C, microtubules, and nucleophosmin (NPM1) [214,215,216,217,218]. These have been used in small clinical trials, in the case of tubercidin with clinical efficacy [219,220,221,222,223,224]. Importantly, differential resistance to these nucleoside analogues has been described in vitro [225]. Pentostatin, a purine analogue inhibiting adenosine deaminase (ADA), is used against hairy cell leukaemia [226]; ADA amplification is a resistance mechanism in vitro [9]. Testing a large set of patient chronic lymphocytic leukaemia (CLL) samples (*n* = 765), low cross-resistance of pentostatin with 2-F-ara-A was reported. Furthermore, in contrast to 2-F-ara-A and 2-CdA, treatment with pentostatin did not induce resistance and is therefore suggested to be a salvage agent for 2-F-ara-A-refractory CLL [227]. Forodesine, a guanosine analogue and inhibitor of purine nucleoside phosphorylase (PNP), exerts T-cell malignancy specific toxicity and does not seem to show cross-resistance to nelarabine [228], even though this has been suggested in resistant cell lines [229].

## 3. Pharmacokinetic Resistance to Nucleobase/Nucleoside Analogues: Delivery

### 3.1. Bioavailability

Nucleoside analogues like cytarabine (ara-C), 5-fluorouracil (5-FU) and gemcitabine (dF-dC) are characterised by low oral bioavailability and high first-pass effects in the liver [230,231]. Hence, these nucleobase/nucleoside analogues are usually administered parenterally to circumvent the digestive tract. Alternatively, lipophilic pro-drugs that require enzymatic conversion to the nucleoside analogue can have much higher oral bioavailability such as capecitabine, pro-drug of 5-FU [232], ara-C ocfosfate [233], pro-drug of ara-C, LY2334737, pro-drug of dF-dC [234], or sapacitabine, pro-drug of CNDAC [235]. On the other hand, a lack of hydrophilicity can critically affect water solubility and thereby achievable plasma concentrations that can be reached even when administering the drug intravenously, as is the case for arabinosylguanine (ara-G). Nelarabine, pro-drug of ara-G, achieves higher water solubility by addition of a methoxy group at the purine 6-carbon, which is subsequently converted into ara-G by ubiquitous adenosine deaminase (ADA). This made clinical application of ara-G possible decades after its discovery [236]. Similarly, fludarabine phosphate is a pro-drug for 2-F-ara-A with much higher water solubility, and is subsequently dephosphorylated by ubiquitous phosphates [237].

### 3.2. Body Compartments as Sanctuaries

The central nervous system (CNS), and other body compartments like the gonads [238,239], maintain a barrier that tightly regulates accessibility of hydrophilic nucleoside analogues [240,241,242,243,244,245]. The efficacy of the blood-brain barrier (BBB) to shield the CNS from antimetabolites is historically best documented by children with ALL who–despite achieving complete remission when treated with antifolates [4]—almost universally relapsed with CNS disease. However, the BBB can be modulated by tumour-secreted signals, as illustrated by Wnt-positive medulloblastoma that has a better response to chemotherapy, as compared to other subtypes of medulloblastoma, due to a disrupted BBB [246]. To achieve clearance of cancer cells in the CNS, the BBB has to be circumvented either by changing the modality to radiation [247], intrathecal application of antimetabolites [248,249], or achieving effective antileukemic CNS concentrations using high-dose systemic regimens of methotrexate [250] or ara-C [251,252], as CNS concentrations of ara-C reach only 40% of plasma concentrations when administered intravenously [230,253].

## 4. Pharmacokinetic Resistance to Nucleobase/Nucleoside Analogues: Stability

Apart from achieving therapeutically relevant peak concentrations of nucleobase/nucleoside analogues in the target tissue, maintenance of effective concentrations over longer periods of time is equally important, especially as these are typically S-phase specific drugs and the cell cycle of a population of tumour cells is asynchronous. However, catabolic enzymes degrade metabolites of nucleobase/nucleoside analogues and critically limit their biological half-life.

### 4.1. Deamination

Adenosine analogues are putative substrates for adenosine deaminase (ADA), which limits the clinical usefulness of vidarabine (ara-A) as an antineoplastic agent if not combined with the ADA inhibitor 2′-deoxycoformycin (pentostatin) [10]. However, 2-CdA, in fact initially developed as an ADA inhibitor, Cl-F-ara-A, and 2-F-ara-A are, due to halogenation of their purine moiety with chlorine (2-CdA and Cl-F-ara-A) and fluorine (2-F-ara-A), intrinsically resistant to ADA [254].

Cytidine deaminase (CDA) in liver and plasma has broad nucleoside promiscuity and deaminates ara-C, dF-dC, decitabine, azacitidine, and CNDAC to the much less potent metabolites arabinosyl-uridine (ara-U), difluorodeoxyuridine (dF-dU) [255], 5′-aza-deoxyuridine (aza-dU) [256], 5′-azauridine (aza-U) [256] and 2′-cyano-2′-deoxy-1-β-d-arabinopentofuranosyluracil (CNDAU) [235], respectively. Germ line polymorphisms of CDA exist that correlate with its enzymatic activity [11,12]. Whereas patients with a high CDA activity suffer from less adverse events following treatment with cytidine analogues, they are also at higher risk for disease progression due to a lack of efficacy as shown for dF-dC with pancreatic cancer [257], decitabine/azacitidine with myelodysplastic syndrome (MDS) [256], and ara-C with acute myeloid leukaemia (AML) [258,259]. A recent study confirmed a significantly higher rate of severe toxicities and death in 58 adult AML patients with lower CDA activity, but also indicated a tendency towards higher response rates [260]. Determination of polymorphisms with known consequences for CDA activity, ex vivo measurement of CDA activity and dose-adjustment following therapeutic drug monitoring (TDM) are warranted to reduce treatment failures due to germ-line resistance. CDA can be inhibited in vivo by the nucleoside analogues tetrahydrouridine (THU) and zebularine [18,19], however, longer exposure times for nucleoside analogues can also efficiently be achieved by protracted intravenous infusion, most extensively studied for ara-C [261], dF-dC [262], and 5-FU [263].

Even though CDA activity is largely absent in the cerebrospinal fluid [264], the half-life of ara-C after intrathecal application remains lower than therapeutically desirable; however, liposomal formulations of ara-C can increase the half-life of ara-C by up to two orders of magnitude [265,266]. Hence, it is clinically not sufficient to use drugs that have toxic efficacy against cancer; its formulation to tailor pharmacokinetics for optimal tumour exposure is at least equally important.

### 4.2. Hydrogenation, Methylation, Deglycosylation

5-FU is readily eliminated to 80% by plasma and liver dihydropyrimidine dehydrogenase (DPD) [267]. It is well studied that DPD activity correlates with the extent of 5-FU toxicity, and underlying polymorphisms in the *DPYD* gene have been characterised in detail [55,56]. As expected, despite a higher risk for toxicity, poor 5-FU metabolisers also benefit from better treatment outcomes [268,269]. Hence, both TDM [267,270] and inhibition of DPD with 5-chloro-2,4-dihydroxypyridine or eniluracil have been suggested to optimise treatment efficacy of 5-FU and capecitabine [57,58,59]. As DPD is also expressed in tumour cells, DPD inhibitors have also been suggested for cancers with high DPD expression [60].

The plasma half-life of TFT is largely determined by the catabolic activity of thymidine phosphorylase (TP, encoded by *TYMP*), removing its deoxyribose sugar moiety. Addition of tipiracil, a potent TP inhibitor, increases the area-under-the-curve for TFT by 37-fold [122]. Combination of TFT and tipiracil as a fixed oral combination has recently obtained FDA approval for metastatic colorectal cancer [123], and is particularly attractive as cross-resistance with other fluoropyrimidines is incomplete [125,126].

Thiopurine S-methyltransferase (TPMT) is a ubiquitously expressed cytoplasmic enzyme that converts the thiopurines 6-thioguanine and 6-mercaptopurine into its less active methylated derivatives [271]. TPMT activity is routinely determined in erythrocytes in a clinical laboratory settings [272,273], and correlates with toxicity [274] and clinical outcomes [275] in leukaemia patients. There is a consistent genotype-phenotype relationship of *TPMT* germ line variants, even though its clinical relevance is debated [119]. Interestingly, the xanthine oxidase inhibitor allopurinol leads to functional inhibition of TPMT, possibly through the production of thioxanthine [120]. Recently, DNA-thioguanine nucleotide concentrations have been suggested as a measure for TDM for thiopurine therapy [276], overcoming resistance by dose-adjustments.

## 5. Tumour-Specific Resistance to Nucleobase/Nucleoside Analogues: Membrane Transport

Nucleoside analogues are principally transported by two membrane transporter families: SLC28, human concentrative nucleoside transporters (hCNT1-3), and SLC29, human equilibrative nucleoside transporters (hENT1-4) [277,278]. hCNTs, with the exception of hCNT3 [279], are expressed in a tissue-restricted manner, mainly in intestinal and renal epithelia, and transport nucleosides in a monodirectional sodium- or hydrogen-dependent manner with specificity for pyrimidines (hCNT1), purines (hCNT2), or both (hCNT3). hENT1-3 are ubiquitously expressed, and hENT4 is mainly present in brain and heart [243,277,280]. Whereas nucleobases like thiopurines and 5-FU can only be transported by hENTs, nucleoside analogues can be transported both by hENTs and hCNTs (an overview of transporter affinity for individual nucleoside analogues can be found here: [278]). As hENTs are bidirectional transporters, the hENT inhibitors nitrobenzylthioinosine (NBMPR) and dipyridamole can inhibit efflux of intracellular nucleoside analogues as demonstrated for 2-CdA [88]. This might be a strategy to increase intracellular exposure to nucleoside analogues when administered sequentially or when influx is primarily mediated by hCNTs [278]. Expression of hENTs has furthermore been positively correlated with in vitro sensitivity to 2-CdA, ara-C and dF-dC [74,75,76,77]. More importantly, hENT1 expression also correlated positively with survival in pancreatic cancer and gallbladder adenocarcinoma patients treated with dF-dC [78,79], as did expression of hENT3 [80]. Similar observations were made in metastatic colorectal cancer treated with trifluridine/tipiracil [81]. When taking into account both hENT1 and hCNT3 expression, high expression of both was strongly associated with improved survival in pancreatic cancer treated with dF-dC, underlining the importance of membrane transport for therapy efficacy [281]. In addition, single nucleotide polymorphisms (SNPs) for nucleoside transporters exist that correlate both with toxicity and therapeutic efficacy of dF-dC in pancreatic cancer [82]. Similar roles of expression levels as well as polymorphisms have been reported for ara-C in the treatment of AML [83,84,85,86,87]. More recently, alternative transport mechanisms for ara-C and dF-dC, involving the SLC22 family, have been described, correlating with overall survival of AML patients treated with ara-C [282].

Purine analogues like 6-TG can be exported by multidrug resistance protein 4 (MRP4), also called ATP-binding cassette transporter 4 (ABCC4) [283], and a SNP in ABCC3 has been correlated with adverse prognosis in AML patients [83]. Similarly, expression of ABCC11 negatively correlated with overall survival in AML [284]. In thiopurine-resistant T-ALL cell lines, down-regulation of hENT2 and hCNT3 was identified [70]. More about the role of ABC transporters for resistance to nucleoside analogues can be found elsewhere [285].

Several approaches exist to overcome dependency on membrane transport proteins for cellular delivery of nucleoside analogues. The elaidic acid ester derivative of ara-C, elacytarabine, is designed to cross the plasma membrane with its lipophilic tail [71] and has been introduced in phase-II and III trials, albeit with modest results [72,73].

## 6. Tumour-Specific Resistance to Nucleobase/Nucleoside Analogues: Intracellular Metabolism

### 6.1. Glycosylation of Nucleobases

Unlike nucleoside analogues, the thiopurine nucleobases and 5-FU need to be glycosylated by means of purine salvage and pyrimidine salvage pathways, respectively. 5-FU can be transformed into 5-F-uridine (5-FUrd) or 5-F-2′-deoxyuridine (5-FdUrd) by uridine phosphorylase 1 (UP1) or TP, respectively [286]. Alternatively, 5-FU can be directly converted into 5-FUrd monophosphate (5-FUMP) by uridine monophosphate synthetase (UMPS, harbouring orotate phosphoribosyl-transferase activity) [286]. In certain cell lines, up-regulation of UP1 leads to increased 5-FU efficacy [136], however, in a cohort of 43 patients with gastric cancer, high expression of UP1 was associated with worse overall survival [287]. In addition, the effects of UP1 inhibitors on 5-FU toxicity are cell-line dependent and can both decrease and increase the efficacy of 5-FU as shown for three different colon cancer cell lines [137], possibly reflecting that the relative contribution mediated by different 5-FU metabolites depends on the cellular background. This is further complicated by the fact that UP1 also catalyses conversion of 5-FdUrd to 5-FU [288]. A Chinese meta-analysis found a positive correlation of TP expression and relapse-free and overall survival of colorectal cancer patients treated with 5-FU [121]. Use of another class of cytotoxic drugs, taxanes, that increases expression of TP, have been suggested as combination therapies to improve 5-FU efficacy [124]. Interestingly, TP has also been reported as a driver of tumour proliferation and metastasis [289], illustrating that nucleoside analogue metabolising enzymes can be double-edged swords, as reported for SAMHD1 [118]. In a 5-FU resistant cell line, UMPS down-regulation was identified [135], whereas experimental overexpression of UMPS increased toxicity of 5-FU in vitro [290,291], and the ratio of UMPS-to-DPD expression has been associated with better overall survival in a Japanese cohort of 5-FU-treated patients with colorectal cancer [292]. High UMPS expression was confirmed to correlate with patient survival in another colorectal cancer cohort [293] as well as in patients with oesophageal squamous cell carcinoma [294]. To the best of our knowledge, associations with *UMPS* polymorphisms have only been reported for toxicity to 5-FU [295], but not for efficacy.

Hypoxanthine guanosine phosphoribosyltransferase (HGPRT, encoded by *HPRT1*) converts the thiopurines 6-MP and 6-TG into 6-thioinosine monophosphate (TIMP) and 6-thioguanosine monophosphate (TGMP), respectively, and TIMP can be further metabolised into thioxanthosine monophosphate (TXMP) and TGMP by inosine monophosphate dehydrogenase (IMPDH) and guanosine monophosphate synthetase (GMPS), respectively [296]. A Dutch study in paediatric patients showed that lower HGPRT activity correlated with poorer prognosis in precursor B-acute lymphoblastic leukaemia (pre-B-ALL), even though no correlation was found with in vitro 6-TG resistance [89]. Another study suggested lower expression of HGPRT to be putatively associated with worse therapy outcome in ALL [90]. Allopurinol, an inhibitor of xanthine oxidase, has been reported or suggested to increase HGPRT activity in patients with inflammatory bowel disease (IBD) and ALL [91,92]. In the myeloid cell line HL-60, acquired mutations in the *HPRT1* gene were detected in half of 6-TG-resistant clones evaluated [297]. Less is known about the role of IMPDH for thiopurine resistance. However, azathioprine (which is extracellularly converted to 6-MP) resistance in one patient with IBD was mediated by an *IMPDH1* promoter mutation negatively affecting transcription [93], suggestive that loss or reduction of IMPDH activity is a possible mechanism of resistance even in cancer patients treated with thiopurines. As for GMPS, its expression was reported to be down-regulated in a thiopurine-resistant T-cell line as compared to the parental cell line [69], but the clinical significance of GMPS warrants further investigation.

### 6.2. Monophosphorylation

Deoxycytidine kinase (dCK) is the principal kinase to activate both pyrimidine and purine nucleoside analogues, however, adenosine kinase, to a lesser extent, has been reported to contribute to purine nucleoside phosphorylation as shown for ara-A [298], although its relevance has been questioned [299]. At least in cell lines and in ex vivo patient-derived primary mantle cell lymphoma cells, downregulation of dCK was not only associated with resistance to ara-C, but also to dF-dC, 2-F-ara-A, and 2-CdA, but not to non-antimetabolic cytotoxic drugs [37]. These findings were confirmed in an independent study [38]. Another study found a correlation of cytotoxic response to decitabine and dF-dC in breast cancer cell lines; interestingly, the same report also identified higher dCK expression in breast cancer tissue from patients with poor outcome as compared to patients with good outcome [39]. Hence, downregulation of dCK, albeit a possible mechanism for resistance to nucleoside analogues ex vivo, might clinically be less relevant as this might be associated with decreased fitness and proliferative potential in vivo. This is corroborated by the finding that reduced dCK expression can lead to sensitisation to corticosteroids in AML, both in cell lines and in patients [40,41]; furthermore, the absence of mutations in *DCK* in relapsed and refractory AML patients argues against that loss of dCK expression in resistant cell lines adequately recapitulates treatment-related resistance [42]. Nevertheless, an earlier study reported that alternative splicing might result in reduced dCK activity in patients with resistant AML [43], and cell lines derived from a patient before and after high-dose ara-C therapy showed reduced dCK expression in two resistant cell clones post therapy [300]. In addition, out of seven patients with ara-C-resistant AML, five showed low activity of dCK [301]. Eventually, polymorphisms in *DCK* can be associated with prognosis in paediatric AML [44] as well as toxicity to ara-C in paediatric ALL [45]. Interestingly, etoposide, a cytotoxic topoisomerase II inhibitor that is routinely combined with ara-C and anthracyclines in paediatric AML treatment, was reported to increase dCK activity [46].

Deoxyguanosine kinase (DGK, encoded by *DGUOK*) activity might marginally contribute to the overall toxicity of purine nucleoside analogues as evidenced in the case of Cl-F-ara-A in dCK-deficient cell lines [52], concomitant reduction of both dCK and dGK in an ara-G-resistant cell line [53], and for 2-CdA and ara-G in dGK overexpression experiments [54]. DGK is–due to its mitochondrial localisation–an interesting candidate to explain S-phase-independent effects of Cl-F-ara-A [302].

Thymidine nucleoside analogues are activated by cytosolic thymidine kinase (TK1). In a colorectal cancer cell line, loss of TK1 expression confers resistance to trifluridine, but retains full sensitivity to 5-FU [34], even though TK1 is critical for phosphorylation of the 5-FU-derivative 2′-deoxy-5-fluorouridine (5-FdU) [303] which–however–can be circumvented by RNR-mediated reduction of 5-FU diphosphate (5-FUDP) to 5-FdUDP. Even though mitochondrial TK2 can phosphorylate ara-C [304], its role for the efficacy of nucleoside analogue therapy remains elusive. On the other hand, nucleosidic inhibitors of TK2 have been suggested as S-phase independent anticancer agents [305].

To overcome deficiency in monophosphorylation as a mechanism of resistance, intracellular delivery of nucleoside analogue phosphates has been suggested. However, due to the high polarity of phosphates and the absence of efficient membrane transport proteins, medicinal chemistry has to be employed to cross the plasma membrane–with similar approaches used to overcome transport deficiency of nucleoside analogues (see above). Aryloxy phosphoramidate triester pro-drugs of nucleoside analogue monophosphates (so called ProTides or pro-tides) allow transporter-independent translocation across the plasma membrane and circumvent the need of phosphorylation by dCK [16,47], and antivirals using this technology have entered the clinic [48]. Phosphoramidates of ara-C and dF-dC have been developed and evaluated pre-clinically, the latter of which having entered a phase-I/II clinical trial [49,50]. Murine L1210 cells resistant to ara-C and dF-dC due to loss of dCK expression were still sensitive to a pro-tide of ara-C [51], being a proof-of-concept for this approach. Other medicinal chemistry strategies of lipophilising nucleoside analogue monophosphates [306] and di- and triphosphates exist, but have not yet entered the clinic due to a variety of problems [101].

### 6.3. Diphosphorylation

UMP/CMP kinase (UCK, encoded by *CMPK1*) is responsible for phosphorylation of cytidine and uridine analogue monophosphates [307,308]. Decreased levels of UCK have been suggested as a mechanism of fluoropyrimidine resistance based on mRNA expression studies in material from colorectal cancers with clinical resistance to 5-FU [132]. In addition, analysis of 80 xenograft models revealed that expression of *CPMK1* is predictive for 5-FU sensitivity [309]. Polymorphisms of *CMPK1* were associated with overall survival of 102 pancreatic cancer patients and lung cancer patients treated with dF-dC [133,134]. Hence, assessment of *CPMK1* status could add information for personalised antimetabolite treatments.

Another monophosphate kinase, mitochondrial UMP/CMP kinase 2, is able to phosphorylate dF-dCMP, but its role for chemotherapy remains to be elucidated [310]. Apart from its suggested role for TFT monophosphate phosphorylation [311], the role of TMP kinase (TMPK) for treatment with nucleoside analogues is largely elusive as its expression–in contrast to *CMPK1*–did not independently correlate with 5-FU sensitivity in xenograft models [309]. Surprisingly, the monophosphate kinase(s) responsible for generation of purine nucleoside analogue diphosphates are not identified nor further characterised.

### 6.4. Triphosphorylation

Nucleotide diphosphate kinase 1 and 2 (NDPK1/2, encoded by *NME1/2*) are supposed to be responsible for catalysis of the last phosphorylation step leading to active triphosphates of virtually all clinically used nucleobase and nucleoside analogues. Whereas polymorphisms in *NME1* correlated with toxicity in AML patients treated with ara-C, no correlation was found with efficacy measures in a cohort of 360 Caucasian patients [99]. A study in Chinese AML patients could not identify a significant correlation of *NME1* SNPs with clinical response, but revealed a significant correlation of a SNP in *NME2* with complete response [100]. Interpretation of these results, however, is difficult, as *NME1* expression has been reported to correlate with poor prognosis in AML [312,313], and NDPK2 harbours tumour suppressor functions [314].

### 6.5. Intracellular Deamination

CDA and ADA are also expressed in tumour cells. In addition, dCMP deaminase (DCTD) deaminates monophosphates of cytidine analogues. Whereas the ADA-mediated block to efficacy of adenosine analogues has been overcome by introduction of ADA-resistant analogues (see above), CDA is a possible driver of resistance to nucleoside analogues [315]. It has been reported that ex vivo toxicity of dF-dC and decitabine correlated negatively with mRNA expression of CDA in primary leukemic blasts from children with AML [316]. A CDA-resistant ara-C-derivative has shown pre-clinical efficacy [317,318], as has a pro-tide derivative of dF-dC [49]. Furthermore, inhibition of CDA with zebularine increased efficacy of ara-C in a murine leukaemia model [19]. However, changes in *CDA* expression in vivo following relapse after ara-C treatments have not been identified [84,300,319]. Nevertheless, it has been hypothesised that *CDA* expression contributes to the intrinsic resistance of solid tumours to ara-C [17]. Polymorphisms of *CDA* exist that correlate with ara-C sensitivity [13], and survival in ara-C-treated AML patients [14] as well as dF-dC-treated ovarian cancer patients [15]. 5-fluorodeoxycytidine has been suggested as a CDA inhibitor, and, following monophosphorylation, as an inhibitor of DCTD [35,36]. In addition, a SNP has been described that affects deaminase activity towards dF-dCMP [320]. However, intratumoural expression of DCTD was not prognostic in a European patient cohort with pancreatic cancer treated with dF-dC [321]. Eventually, it should be noted that even deaminated nucleoside analogues might harbour some activity, even though this is not well studied [322].

### 6.6. Dephosphorylation of Monophosphates

The pool of active metabolites of nucleobase/nucleoside analogues can be limited by the action of dephosphorylating enzymes, with cytosolic 5′-nucleotidase I, II and III (cN-I/II/III encoded by *NT5C1/2/3*) cleaving monophosphates of nucleoside analogues. Among the cytosolic nucleotidases, cN-II has been studied most extensively. Overexpression of *NT5C2* leads to decreased sensitivity to ara-C, dF-dC, 2-F-ara-A, Cl-F-ara-A and 2-CdA [20,21]. The mRNA expression levels of *NT5C2* were associated with adverse clinical outcome in adult AML patients treated with ara-C [22], and, when combined with expression levels of *DCK*, negatively correlated with accumulation of ara-CTP in primary paediatric AML cells, and ara-C sensitivity in AML cell lines [23,24,25]. In lung cancer patients treated with dF-dC, protein expression of cN-II was significantly correlated with overall survival [26]. Gain-of-function mutations in *NT5C2* can be found in relapsed ALL patients and leads to 6-mercaptopurine resistance in mouse models of ALL [20,28,29,30,31]. Eventually, polymorphisms of *NT5C2* that correlate with decreased survival in paediatric and adult AML have been described [14,32].

*NT5C3* expression and polymorphisms have been associated with in vitro efficacy of dF-dC and ara-C [323], and a SNP correlating with *NT5C3* expression was associated with the rate of complete response in AML patients treated with ara-C [33]. Overexpression of *NT5C1* conferred resistance to ara-C, 2-CdA, dF-dC, 2-F-ara-A and 5-FU in cell lines [27]. Currently, no inhibitor of cytosolic nucleotidases exists, but lipophilic pro-drugs of nucleoside analogue diphosphates and triphosphates could circumvent the step of monophosphorylation and thereby deprive nucleotidases of their substrates [101].

### 6.7. Dephosphorylation of Triphosphates

For many nucleobase/nucleoside analogues, it is the triphosphate metabolite that is responsible for clinical efficacy. Thus, enzymes which directly modulate the intracellular triphosphate pools, of which there are many [324], can play critical roles in modulating treatment efficacy.

Expression of the dUTP diphosphatase, dUTPase, negatively correlates with therapy response to 5-FU, which could either be mediated by reduced dUTP accumulation following TS inhibition [63], or by reduced accumulation of the 5-FU active metabolite 5-F-dUTP [64], see above. Similarly, overexpression of dUTPase reduces the efficacy of decitabine by reducing the pools of 5-aza-dCTP and 5-aza-dUTP, which also leads to an increase in 5-aza-dUMP possibly resulting in additional inhibition of TS [325,326]. Consistently, inhibitors of dUTPase have been reported to increase the efficacy of 5-FU [65,66,67]. A dual inhibitor of DPD and dUTPase has been shown to increase the antineoplastic effects of capecitabine towards a human breast cancer xenograft in mice [68]. Interestingly, pre-treatment of a colon-cancer cell line with oxaliplatin decreased dUTPase expression and increased sensitivity to 5-FU [327]. A related pyrophosphatase, dCTPase (also called DCTPP1), has been shown to reduce the efficacy of dF-dC and decitabine in leukemic cell lines [63,328], and a number of pre-clinical inhibitors for dCTPase have been reported [328,329].

The nucleotide diphosphatase NUDT15, a member of nudix hydrolase family, possesses activity towards a selection of canonical nucleotides and their oxidised variants, and has been proposed to have a role in dNTP pool sanitation [324]. A missense mutation of *NUDT15*, R139C, in patients of Asian descent, was found to significantly correlate with thiopurine intolerance in ALL and IBD patients [330,331,332]. Shortly after this finding, NUDT15 was demonstrated to possess hydrolytic activity towards the active triphosphate metabolites of thiopurines, 6-thio-(d)GTP [98], and accordingly, control cytotoxicity of this analogue in cancer cell models [333,334]. The NUDT15 R139C variant was shown to affect protein stability [334], and in line with this, patients with this mutation, and others resulting in defective NUDT15, had increased levels of thiopurine active metabolites and accompanying toxicity [333]. Personalised thiopurine therapy could circumvent these problems, and with this in mind, a recent study utilised a novel *NUDT15* knockout mouse model to demonstrate the feasibility of *NUDT15* genotype-guided dose individualisation. This study effectively showed that, using this strategy, drug toxicity could be prevented while maintaining therapy efficacy [335].

Inosine triphosphatase (ITPA) is a pyrophosphatase regulating sanitation of the endogenous non-canonical (deoxy)nucleotide triphosphates (deoxy)inosine, and (deoxy)xanthosine triphosphate, that can also use 6-thio-ITP as a substrate [336,337,338]. Polymorphisms in *ITPA* have been described to correlate with intolerance to mercaptopurine treatment in IBD [339], even though these results have been questioned in a later meta-analysis [340]. As 6-thio-ITP is a direct substrate for TPMPT, reducing the thiopurine nucleotide pool that can be used as a substrate for DNA polymerases, activity of ITPA shifts metabolism of thiopurines towards metabolites that can be incorporated into DNA. Accordingly, an *ITPA* polymorphism has been shown to correlate with levels of methylated thiopurine metabolites in 66 children with ALL [94], levels of 6-TG incorporation into DNA in 132 ALL patients, and is a factor highly likely to influence outcome of ALL patients treated with thiopurines [95]. Furthermore, this *ITPA* SNP was associated with toxicity [96] and treatment outcome in a British ALL cohort [97].

In 2015, it was shown that the dNTP triphosphohydrolase SAMHD1—a protein heavily involved in the evolution the human immunodeficiency virus [341], and the only known eukaryotic enzyme that can cleave nucleotide triphosphates to nucleosides and inorganic triphosphate—is able to hydrolyse Cl-F-ara-ATP [342]. We and others have subsequently shown that other nucleoside analogue triphosphates are substrates or substrate candidates for SAMHD1, in particular ara-CTP, ara-GTP, 5-aza-dCTP, and ara-ATP [117,118,343,344,345]. Furthermore, we could show that expression levels of SAMHD1 correlate with event-free and overall survival, but, importantly, not with complete remission, of adult and paediatric AML patients treated with ara-C [117,118]. We have suggested the use of the viral protein X (Vpx) as a strategy to inhibit SAMHD1 activity towards nucleoside analogue triphosphates [117], and the development of in vitro and cell-active small molecule inhibitors of SAMHD1 is underway [346,347,348].

## 7. Tumour-Specific Resistance to Nucleobase/Nucleoside Analogues: Pharmacodynamics

Overexpression of the targets of nucleoside analogue active metabolites is one mechanism to mitigate and escape toxicity, as evidenced extensively for RNR in cell lines [103,104,105,106], mouse models of colon cancer [107] and patient material [108,109,110,111,112,113,114]. Furthermore, polymorphisms of the *RRM1* promoter correlated with clinical response to dF-dC in non-small cell lung cancer patients [115]. Inhibitors of the MEK-ERK signalling pathway have been shown to increase dF-dC sensitivity by reducing RNR expression [349]. Analogous to RNR, overexpression of *TYMS*, the gene encoding TS, can lead to resistance to TS inhibitors, in particular fluoropyrimidines, both in vitro [116,127,128,129] and in patients [130]. Histone deacetylase inhibitors have been shown to reduce *TYMS* expression and thereby synergise with fluoropyrimidines [128,350]. Along these lines, increased activity and expression of DNMT1 is a possible mechanism for decitabine resistance whereas *DNMT3A* mutations lead to increased efficacy of decitabine [61,102]. Also increased activity of PPAT, inhibited by thiopurine monophosphates, is a possible mechanism of resistance to these groups of nucleobase analogues [131]. Future studies will have to elucidate the contribution of expression levels of DNA glycosylases/BER and DNA mismatch repair [351] components to fluoropyrimidine resistance.

## 8. Tumour-Specific Resistance to Nucleobase/Nucleoside Analogues: Tumour Biology

Eventually, even if high levels of active metabolites can be achieved intracellularly, and even if the pharmacodynamic targets can be sufficiently antagonised, the efficacy of nucleoside and nucleobase analogues is not guaranteed. A recent study showed that ara-C-resistant cells can actually incorporate much higher amounts of an ara-C-derivative, AzC, that is amenable for super-resolution microscopy, into DNA as compared to ara-C-sensitive cells without leading to cell death. Instead, AzC incorporation led to highly stable stalled replication forks, and DNA replication was resumed once AzC was removed from the medium. Furthermore, AzC was detectable in daughter-cell DNA [352]. Hence, modulation of down-stream effectors responding to stalled replication forks and DNA-damage are likely to be responsible for ara-C resistance in this context. Tumour evolution can be driven by the loss of tumour suppressors. Not surprisingly, loss of factors that control DNA integrity limit cytotoxicity of nucleoside analogues, as shown for p53 [353,354] and Schlafen 11 (SLFN11) [355]. Strategies to reactivate p53 or target mutant p53 [356,357] and SLFN11 via histone deacetylase or EZH2 inhibition are under development [358,359].

## 9. Clinical Resistance to Nucleobase/Nucleoside Analogues: Therapy-Limiting Toxicity

Nucleoside analogues are S-phase, but not tumour-specific drugs, and will therefore exert toxic effects in proliferative normal tissues. In vitro, resistance to a nucleobase or nucleoside analogue is usually a relative measure, indicated by highly increased concentrations to achieve killing of cancer cells in comparison to sensitive cancer cells. Translated into the clinic, this usually means that these concentrations could not be achieved in vivo due to therapy-limiting toxicities. In other words, clinical resistance can be interpreted as a therapeutic window of zero (or below), which means that concentrations necessary to cure the cancer cannot be reached due to unacceptable toxicities. Comparing expression levels of key factors involved in transport, metabolism and drug effector functions in tissues at risk with the tumour, informs about the breadth of the therapeutic window. Capecitabine is a prime example where differential expression of the activating enzyme TP correlates with an increased therapeutic window due to selective accumulation of 5-FU [360].

## 10. Conclusions

Curing cancer with chemotherapy has been possible since the 1960’s, and the necessity of combination chemotherapy is evident. Nucleobase and nucleoside analogues are an important class of anti-cancer drugs for solid and haematological malignancies. Understanding the clinical and molecular determinants of chemotherapeutic efficacy is paramount for further treatment improvements. As lack of efficacy can be caused at a pharmacokinetic, a metabolic and a pharmacodynamic level, and be further complicated by the underlying intrinsic tumour biology, a multitude of possible treatment modulations, or change of therapy modality altogether, exist. However, understanding the net consequence of patient- and tumour-specific predictors of therapy outcome is an immense task. We nevertheless believe that nucleoside analogues are ready to enter the era of precision medicine [361].

## Figures and Tables

**Table 1:** Overview on pharmacokinetic and pharmacodynamic factors for nucleobase/nucleoside analogues, resistance mechanisms, and strategies of re-sensitisation.

Protein			Properties	Resistance Mechanism	Re-Sensitisation
Name	Abbrev.	Gene			
**Adenosine deaminase**	ADA	*ADA*	Deamination of adenosine analogues	ADA amplification [9]	ADA inhibitors (pentostatin) [10], ADA-resistant nucleosides
**Cytidine deaminase**	CDA	*CDA*	Deamination of (deoxy)cytidine analogues	Polymorphisms [11,12,13,14,15], Overexpression [16,17].	CDA inhibitors [18,19]
**Cytosolic 5′-nucleotidase I, II and III**	cN-I/II/III	*NT5C1–3*	Dephosphorylation of nucleoside analogue monophosphates	Over-expression [20,21,22,23,24,25,26,27], gain-of-function mutations [20,28,29,30,31], polymorphisms [14,32,33]	
**Cytosolic thymidine kinase**	TK1	*TK1*	Monophosphorylation of thymidine analogues	Loss of TK1 expression confers resistance to trifluridine [34]	
**dCMP deaminase**	DCTD	*DCTD*	Deaminates monophosphates of cytidine analogues		DCTD inhibitor [35,36]
**Deoxycytidine kinase**	dCK	*DCK*	Monophosphorylation of pyrimidine and purine nucleoside analogues	Downregulation [37,38,39,40,41,42], Alternative splicing resulting in reduced dCK activity [43], Polymorphisms [44,45]	Etoposide to increase dCK activity [46], Pro-tide chemistry [16,47,48,49,50,51]
**Deoxyguanosine kinase**	dGK	*DGUOK*	Monophosphorylation of purine nucleoside analogues	Activity/expression [52,53,54]	
**Dihydropyrimidine dehydrogenase**	DPD	*DYPD*	Reduction of uracil and thymine analogues	Polymorphisms [55,56]	DPD inhibitors 5-Chloro-2,4-dihydroxypyridine, Elinuracil [57,58,59,60]
**DNA methylatransferases**	DNMTs	*DNMT1, DNMT3A, DNMT3B, DNMT3L*	Methylation of 5′ cytosine in GpC dinucleotides	Increased activity/expression of DNMT1 [61]	Change to Zebularine [62]
**dUTP diphosphatase**	dUTPase	*DUT*	Dephosphorylation of dUTP analogues	Overexpression [63,64]	dUTPase inhibitors [65,66,67,68]
**Guanosine monophosphate synthetase**	GMPS	*GMPS*	Conversion of TXMP to TGMP	Downregulation [69]	
**Human concentrative nucleoside transporters 1–3**	hCNT1–3	*SLC28A1*–*3*	Unidirectional membrane transport	Downregulation [70]	Lipophilic modifications [71,72,73], pro-tide chemistry [16,47,48,49,50,51]
**Human equilibrative nucleoside transporters 1–4**	hENT1–4	*SLC29A1–4*	Bi-directional membrane transport	Low expression [74,75,76,77,78,79,80,81], Polymorphisms [70,82,83,84,85,86,87]	Efflux inhibitors [88], Lipophilic modifications [71,72,73], Pro-tide chemistry [16,47,48,49,50,51]
**Hypoxanthine guanosine phosphoribosyltransferase**	HGPRT	*HPRT1*	Converts thiopurines 6-MP and 6-TG into 6-thioinosine monophosphate (TIMP) and 6-thioguanosine monophosphate (TGMP)	Decreased activity/expression [89,90]	Allopurinol to increase HGPRT activity [91,92]
**Inosine monophosphate dehydrogenase**	IMPDH	*IMPDH1*	Conversion of TIMP to thioxanthosine monophosphate (TXMP)	Loss or reduction of activity [93]	
**Inosine triphosphatase**	ITPA	*ITPA*	Regulates sanitation of the endogenous non-canonical (deoxy)nucleotide triphosphates (deoxy)inosine and (deoxy)xanthosine triphosphate	Polymorphisms [94,95,96,97]	
**Nucleotide diphosphatase NUDT15**	NUDT15	*NUDT15*	Dephosphorylation of thiopurine triphosphates	High expression [98]	
**Nucleotide diphosphate kinase 1 and 2**	NDPK1/2	*NME1/2*	Phosphorylation of nucleoside analogue diphosphates	Polymorphisms [99,100]	Lipophilising diphosphate analogues [101]
**Phosphoribosyl pyrophosphate amidotransferase**	PPAT	*PPAT*	Purine biosynthesis	Increased activity of PPAT [102]	
**Purine nucleoside phosphorylase**	PNP	*PNP*	De-glycosylation of guanosine/inosine analogues		
**Ribonucleotide reductase**	RNR	*RRM1, RRM2, RRM2B*	Reduction of nucleoside diphosphates (NDPs) to deoxy-NDPs (dNDPs)	Overexpression [103,104,105,106,107,108,109,110,111,112,113,114], Polymorphisms [115]	MEK-ERK inhibitors increase dF-dC sensitivity by reducing RNR expression [116]
**SAM and HD domain protein 1**	SAMHD1	*SAMHD1*	Dephosphorylation of dNTP analogues	High expression [117,118]	Use of viral protein X to inhibit SAMHD1 [117], Small-molecule inhibitors [103,104,105]
**Thiopurine S-methyltransferase**	TPMT	*TPMT*	Methylation of thiopurines	Polymorphisms [119]	Xanthine oxidase inhibitors (allopurinol) [120]
**Thymidine phosphorylase**	TP	*TYMP*	Glycosylation of 5-FU, De-glycosylation of thymidine analogues	Low expression in tumour tissue (for 5-FU treatment) [121], High plasma activity (for TFT treatment) [122,123]	Taxanes to increase expression (for 5-FU treatment) [124], Inhibitor tipiracil (for TFT treatment) [122,123,125,126]
**Thymidylate synthase**	TS	*TYMS*	Reductive methylation of deoxyuridine monophosphate (dUMP) to deoxythymidine monophosphate (dTMP)	Overexpression [116,127,128,129,130]	Histone deacetylase inhibitors (HDACi) reduce *TYMS* expression and synergise with fluoropyrimidines [130,131]
**UMP/CMP kinase**	UCK	*CMPK1*	Phosphorylates cytidine and uridine analogue monophosphates	Downregulation [132], Polymorphisms [133,134]	Lipophilising diphosphate analogues [101]
**Uridine monophosphate synthetase**	UMPS	*UMPS*	Conversion of 5-FU to 5-FUMP	Downregulation [135]	
**Uridine phosphorylase 1**	UP1	*UPP1*	Glycosylation of 5-FU, De-glycosylation of 5-FdUrd	Expression [136]	UP1 inhibitors (cell-line dependent effects) [137]
